# Who Is Doing the Housework in Multicultural Britain?

**DOI:** 10.1177/0038038516674674

**Published:** 2016-12-12

**Authors:** Man-Yee Kan, Heather Laurie

**Affiliations:** University of Oxford, UK; University of Essex, UK

**Keywords:** domestic division of labour, ethnicity, gender

## Abstract

There is an extensive literature on the domestic division of labour within married and cohabiting couples and its relationship to gender equality within the household and the labour market. Most UK research focuses on the white majority population or is ethnicity ‘blind’, effectively ignoring potentially significant intersections between gender, ethnicity, socio-economic position and domestic labour. Quantitative empirical research on the domestic division of labour across ethnic groups has not been possible due to a lack of data that enables disaggregation by ethnic group. We address this gap using data from a nationally representative panel survey, *Understanding Society*, the UK Household Longitudinal Study containing sufficient sample sizes of ethnic minority groups for meaningful comparisons. We find significant variations in patterns of domestic labour by ethnic group, gender, education and employment status after controlling for individual and household characteristics.

## Background

There is an extensive UK and international literature on the domestic division of labour within married and cohabiting couples and its relationship to gender equality within the household and the labour market (Baxter, 2002; Bianchi et al., 2000; Kan, 2008; Lyonette and Crompton, 2015). With increasing numbers of women entering the labour market in recent decades, the proportion of women’s housework time relative to men’s has been decreasing gradually but women still undertake the bulk of housework (Kan et al., 2011). The decline in women’s domestic work time focuses primarily on routine housework such as cleaning, cooking and doing the laundry. Men have increased their participation mainly on non-routine domestic work such as grocery shopping and home-repairs. There is limited evidence of gradual change or ‘lagged adaptation’ by men in response to women’s paid employment (Gershuny et al., 1994) although Baxter (2002) finds that the reducing gender gap in the share of housework hours is not due to men increasing their housework hours but rather to women reducing the hours they spend on housework. Despite significant liberalisation in gender attitudes this does not necessarily translate into changes in behaviour which remain gendered (Crompton et al., 2005). This is attributed in part to women working fewer hours to allow them to combine domestic tasks with paid employment alongside the increasing intensification of work for many men, something which may restrict men’s ability to contribute to domestic labour even if they wished to. We know little about how these patterns vary by ethnic group.

Most UK research on the domestic division of labour focuses on the white majority population or is ethnicity ‘blind’, effectively ignoring potentially significant associations between gender, ethnicity, socio-economic position and domestic labour. We have limited information about variations in domestic labour patterns and inequalities within the household across ethnic groups that may be associated with the labour market status of specific groups and their socio-economic characteristics. While there is considerable evidence of the disadvantaged labour market position and risks of relative poverty of ethnic minority groups in the UK (e.g. Fisher and Nandi, 2015), quantitative empirical research on the domestic division of labour across ethnic groups has not been possible, largely due to a lack of data that enables disaggregation by ethnic group and an examination of the heterogeneity in patterns of domestic labour across ethnic groups.

We address this gap using data from a nationally representative panel survey, *Understanding Society*, the UK Household Longitudinal Study. The survey contains sufficient sample sizes of ethnic minority groups in the UK to enable meaningful comparisons across ethnic minority groups and with the white British majority population. We focus on the extent to which domestic arrangements are egalitarian as measured by the number of hours and share of time spent on domestic tasks by men and women in married or cohabiting couples. We examine how hours spent on domestic labour vary by ethnic group accounting for education, employment and individual and household characteristics. The aim of the article is to provide the first nationally representative evidence on ethnicity and the domestic division of labour, increasing our understanding of the intersections between gender, ethnicity and household labour in married and cohabiting couples in the UK.

### Theoretical Perspectives

Within the literature, there have been three main theoretical approaches to understanding gender inequalities in the domestic division of labour based on: (1) time availability; (2) resource bargaining; and (3) gender roles and ‘doing gender’. Theories of time availability and resource bargaining assume rationality in how resources are allocated. The time availability perspective is associated with neo-classical economic models of household behaviour where men and women are assumed to cooperate to maximise their joint utility and comparative advantage by specialising in the labour market or domestic work respectively (Becker, 1991 [1981]). Hakim (2002) argues that the gendered patterns of behaviour between paid employment and the domestic sphere are explained by what she terms ‘preference theory’ where only a minority of women ‘choose’ to be work-centred and prioritise their career over their family role. This approach has been criticised for assuming women have unconstrained ‘choices’ with longitudinal evidence suggesting that choices made depend on how constraints differentially affect women at different points in their lives, for example, following childbirth (Kan, 2007; McRae, 2003).

The resource-bargaining approach also emphasises the role of rationality but argues both men and women aim to maximise their individual welfare rather than their joint utility (e.g. Lundberg and Pollak, 1996). In this theory domestic work time is determined by relative income levels. As men are on average paid more than women, men on average do less housework because they contribute a larger part of the family income. This perspective assumes both husbands and wives tend to avoid housework so a rise in women’s contribution to the family income will reduce their housework time and increase their husbands’ housework time (Bianchi et al., 2000). There is some empirical support for the resource bargaining approach as women who contribute more income to the household have been found to have greater negotiating power within the relationship leading to more egalitarian domestic arrangements (Lyonette and Crompton, 2015).

Time availability and resource bargaining theories both neglect the role of gender norms and identity in determining domestic arrangements. A fairly crude interpretation is that men and women ‘do gender’ through mainly devoting themselves to the public or private sphere respectively (e.g. Brines, 1994). Women display their feminine identity through doing housework and looking after the family (DeVault, 1990) while men obtain their sense of masculine identity by taking up the ‘breadwinner’ role (Nolan et al., 2000). While gender roles have been somewhat blurred with the increasing participation of women in the labour market alongside a shift to less traditional gender attitudes (Crompton et al., 2005), ‘doing gender’ is also expressed through domestic practices. The home is the domain where women’s unpaid domestic labour is exploited and where public discourses on gender norms and family ideals are actively contested. Increasingly demanding standards for housework have also been set, for example, by advertisements of modern domestic appliances (Shove and Southerton, 2000) and expectations of women creating the perfect home through housekeeping magazines (Hand and Shove, 2004; Martens and Scott, 2005).

Housework may also become a symbolic enactment of gender identities when gendered expectations are violated, for example when the woman earns significantly more than her partner. Some studies have found a curvilinear relationship, rather than a linear one, between women’s relative earnings and their time and share of housework, indicating that women may ‘do gender’ by undertaking housework even when they are earning more or working longer hours than their partner (Bittman et al., 2003; Greenstein, 2000). Others have found no gender display effect (Evertsson and Nermo, 2004) and Kan (2008) reported a linear relationship between relative income and housework hours when paid work hours are taken into account.

### Intersectionality of Gender and Ethnicity

The primary concern in these theoretical approaches is gender with potentially important intersections between gender, ethnicity and inequalities in domestic labour not considered. Intersectionality as a framework to understand the vulnerabilities of women of colour in terms of economic dislocation, lack of access to education and violence against women has emerged in recent years in US feminist campaigns against racism and social inequality (Crenshaw, 2015). Intersectionality between gender and ethnicity has also been examined in the context of the labour market (Brah, 1993; Mintz and Krymkowski, 2011). There are two main aspects to intersectionality. First, ethnic minority women may face double penalties in the labour market due to their gender as well as ethnicity, and hence the two disadvantages are accumulated. Second, there may be different factors and mechanisms determining the structures of inequalities by gender and ethnicity and there may be interactions among these factors and mechanisms. Therefore gender inequalities may exhibit in different forms among ethnic groups depending on structural factors as well as individual characteristics. Studies of intersectionality argue against a general theory of gender for analysing paid work and highlight the importance of studying the intersections between gender and ethnicity to understand patterns of labour market participation and inequalities due to a combination of gender and ethnicity in labour market position, wages and occupations. In relation to the domestic division of labour we suggest that the factors explaining the domestic division of labour *across* ethnic groups may not be the same for men and women while the factors explaining differences *between* men and women may not be the same across ethnic groups. Within the overarching framework of intersectionality we are therefore concerned to examine the degree to which both gender and ethnicity structure inequalities in the domestic division of labour.

### Ethnicity, Immigration, Diversity and Disadvantage

In recent decades the UK has experienced a significant increase in immigration leading to a diverse and multicultural society. The largest immigrant groups are from post-colonial countries including the Indian sub-continent and the Caribbean with European Union immigrants arriving more recently. The immigrant and ethnic minority population is diverse in terms of the motives driving their migration decision, country of origin, cultural background, education and skills and duration since arrival in the UK (Luthra et al., 2014). There may also be differences between first and second generation immigrants due to language and other barriers to integration for first generation immigrants. While immigrants will bring their cultural norms, values and expectations about family life from their country of origin it might be expected that those born and/or educated in the UK may adopt a mix of norms and values from their culture of origin and from the UK (Nandi and Platt, 2014).

Ethnic minority groups in the UK suffer from multiple disadvantages relative to the white British population across a range of socio-economic outcomes (Fisher and Nandi, 2015) and experience persistent disadvantage in the labour market (Catney and Sabater, 2015). Gender pay gaps for Pakistani, Bangladeshi and black Caribbean women are greater than for white British workers because these women tend to work in low-skilled jobs (Brynin and Guveli, 2012), ‘ethnic penalties’ that are not due to differences in education or other individual characteristics (Longhi et al., 2013).

Given the known associations between women’s employment patterns and the domestic division of labour (Kan, 2008) we might expect the domestic division of labour for ethnic minority women in the UK to be associated with employment status. Women from ethnic minority groups have higher unemployment rates than white British women and are more likely to be in low paid, low status jobs, particularly those from a Bangladeshi or Pakistani background (Catney and Sabater, 2015). Cultural and language barriers and a lack of knowledge about the UK labour market can lead to migrants taking low paid, low skill jobs and even migrants highly qualified in their country of origin may find it problematic to establish themselves in the UK labour market due to a lack of transferability of qualifications (Green, 2013). We might expect second generation immigrants educated in the UK will integrate more successfully into the labour market despite the ethnic penalties which persist. These differing experiences within the labour market are likely to have an impact on household labour and gender roles within the household.

### Previous Research

The majority of research into the domestic division of labour and ethnicity is US based. Some studies find African-American men are more traditional in terms of women’s primary role as homemakers while some find African-Americans have more egalitarian attitudes than white Americans (Kane, 2000). Using US time-use data, Sayer and Fine (2011) find women from an Asian or Hispanic background spend more time on domestic work than black or white women but no differences in men’s time spent on domestic work across ethnic groups. They suggest differences in domestic labour across ethnic groups may be due to cultural differences in how domestic tasks are defined producing variations in egalitarian attitudes and behaviours. Ting et al. (2015) compared the time spent on domestic labour across different immigrant and ethnic groups using Australian panel data. While all women spend more time on housework than men, they find significant gender gaps in housework hours by ethnic group that are not explained by observable socio-economic characteristics. Ting et al. (2015) conclude theoretical approaches used to understand the gendered division of household labour in majority populations may not be appropriate across all ethnic groups where distinct meanings or cultural definitions of what constitutes domestic work may differ.

In the UK most research has been qualitative, focusing on South Asian communities. Archer (2002) argues institutional racisms and sexism may play an important part in influencing Asian girls’ education and employment aspirations which cannot be constructed as entirely due to cultural background. In a study of young Pakistani women Brah (1993) found most were in favour of women’s right to paid employment but their position in the labour market was due to a complex interplay of factors. These included opportunities in the local labour market, cultural ideologies about women and paid work, the role of education in mediating job aspirations, religion and racism.

Given the paucity of quantitative evidence for the UK we have three main research questions and associated hypotheses:

1. How does the domestic division of labour and share of time spent on domestic tasks vary by ethnic group in the UK? We expect the domestic division of labour varies by ethnic group and by gender, given the differences in migration history and labour market experiences among ethnic groups.
2. How is the domestic division of labour mediated by educational attainment, employment status and gender attitudes? Following the perspective of intersectionality, we expect that differences in the patterns of domestic division of labour among ethnic groups are to a certain extent due to differences in their educational attainment, employment status and gender attitudes. Following the predictions of resource bargaining theory and time availability approaches, we expect to find that housework hours and housework share are negatively associated with employment status and educational attainment.
3. Are there differences between first and second generation immigrants in patterns of domestic labour? Immigration experience is another key factor influencing the intersectionality between gender and ethnicity. We expect those who were born in the UK or arrived in the UK before age 12 (‘one-and-a-half’ generation immigrants) to have different domestic division of labour arrangements compared to first generation immigrants, because these individuals would have spent most or some of their formative years in the UK education system and been more widely exposed to British cultural norms.

## Data, Key Measures and Analysis Approach

The data are from *Understanding Society*, the UK Household Longitudinal Study. *Understanding Society* is an annual household panel survey of individuals in 40,000 households in the UK at wave 1 (2009/2010) where individuals aged 16 and over are interviewed annually. The *Understanding Society* sample is a general population probability sample representing the UK population but also includes a substantial ethnic minority boost sample (EMB). The EMB is designed to provide additional samples of 1000 individuals in each of five main ethnic minority groups – Indian, Pakistani, Bangladeshi, black African and black Caribbean and includes first, second and third generation immigrants. Other ethnic minority groups including Chinese, Middle Eastern and EU migrants are not oversampled but were included in the boost sample when identified during screening (Berthoud et al., 2009).

The analytic sample includes heterosexual married or cohabiting men and women of working age (16 to 64 years old). We pool cases from wave 2 (2010/2011) and wave 4 (2012/2013) when questions on domestic labour and gender-role attitudes were asked. Table 1 gives the analytic sample size for couples by ethnicity and gender. Overall, 21 per cent of respondents are in an ethnic group other than white British with the largest groups being white Other, Indian, Pakistani and Bangladeshi reflecting the main sending countries and post-war immigration patterns.

**Table 1.** Analytic sample: distribution of ethnic groups by gender (married and cohabiting respondents aged 16–64 years: waves 2 and 4, *Understanding Society*, unweighted data).

Ethnic group	Men	Women	Total
White British	10,953	11,456	22,409
White Irish	169	194	363
White Other	400	579	979
Indian	557	585	1142
Pakistani	378	389	767
Bangladeshi	320	319	639
Chinese	61	91	152
Other Asian	164	254	418
Black Caribbean	152	166	318
Black African	268	246	514
Mixed background	171	208	379
Other ethnic group	149	166	315
Total	13,742	14,653	28,395

### Key Measures

Ethnic group was self-reported by individuals using the 2011 UK Census question wording and categories. Respondents could self-identify as being of Mixed Asian/black African/black Caribbean ethnic background and we maintain this as a separate group for analysis. Those who identify themselves as ‘Mixed’ may identify with both their origin ethnicity and with being British and have different behaviours compared to those who identify primarily with one ethnic group. We also separate the ‘white’ group by whether white British, white Irish or white Other to enable examination of potential differences between these groups. The white Other group includes migrants from countries such as Australia, Canada or the USA as well as EU migrants self-identifying as ‘white’. Information on own and parents’ country of birth and dates of arrival in the UK provide information on whether first or second generation immigrants.

Questions on the domestic division of labour were asked of married and cohabiting individuals who reported how many hours they spend in an average week on housework such as cooking, cleaning, washing and ironing. While stylised survey estimates on hours of housework are not ideal when compared to more accurate time-diary estimates, the reporting errors are largely random (Kan and Pudney, 2008). Therefore they do allow the construction of a variable to indicate the total share of housework time for each couple member. One potential limitation of the domestic labour measures is they may not be interpreted in the same way across all ethnic groups. We have no way of assessing this in this study but others have suggested the definition of what constitutes domestic labour may vary across groups making direct comparisons problematic (Sayer and Fine, 2011; Ting et al., 2015). A future qualitative study to understand potential differences of interpretation could shed some light on this issue.

*Understanding Society* interviews both couple members allowing comparisons of spouse’s responses. Information on educational qualifications and details of employment, income and family status are collected allowing variables on the couple’s joint educational and employment status to be derived.

Respondents were asked a series of gender-role attitudes questions in a self-completion section of the survey where they ranked statements on a five-point scale from ‘Strongly Disagree’ to ‘Strongly Agree’. The statements used for this analysis are included on many large-scale surveys and include:

- A pre-school child is likely to suffer if his or her mother works.
- All in all, family life suffers when the woman has a full-time job.
- Both the husband and wife should contribute to the household income.
- A husband’s job is to earn money; a wife’s job is to look after the home and family.

Potential measurement problems such as social desirability effects should be minimised by the self-completion mode as people could respond without the interviewer being aware of their responses. These questions are well validated and widely used in the literature although we have no means of assessing whether the questions are interpreted consistently across all ethnic groups. The responses were recoded to derive an overall gender attitudes score ranging from a possible −8 to +8 with ‘0’ being neutral. A negative score indicates less traditional gender-role attitudes and a positive score more traditional gender attitudes.

### Analysis Approach

We carry out a cross-sectional analysis on the pooled sample from waves 2 and 4 of *Understanding Society* (2010/2011 and 2012/2013). We report descriptive results on the associations between ethnic group and the number of hours spent on housework per week by gender and the share of domestic labour within couples. Multivariate OLS regressions examine the relationship between ethnic group and domestic labour after holding individual and other characteristics constant. We then include interaction effects between ethnic group and education, employment and gender-role attitudes to examine within group effects. As the analysis is predominantly descriptive and cross-sectional, we use survey weights to account for survey design, unequal probabilities of selection and non-response. Robust standard errors are used to adjust for individual clustering in the sample. Including gender-role attitudes in the regression models is somewhat problematic as these are likely to be endogenous predictors of patterns of domestic labour. For this reason we run the models separately with and without gender-role attitudes. The interpretation of the main results remains unchanged even though the magnitudes of some estimates vary once gender-role attitudes are included.

Our main dependent variables are (1) usual weekly housework hours and (2) the share of housework between couple members. The main predictors are own and spouse’s education level (whether has a degree or not), own and spouse’s employment status (whether in paid employment or not) and immigrant generation (whether born in the UK or arrived before the age of 12 years or not).

The control variables include age, whether spouse is from the same ethnic background, marital status (cohabiting vs married), monthly household income, number of dependent children and the survey year. A variable indicating the ethnicity of the spouse is included because both the respondent’s and the spouse’s ethnicity may play a role in the domestic division of labour. Overall, 11 per cent of individuals had a partner from a different ethnic background and 26 per cent of non-white British had a spouse from a different ethnic group so not all couples are homogeneous in terms of a common ethnic background. Health status is excluded as it was not significant in the models. Religious affiliation is not included as this is heavily confounded with ethnic group and the sample sizes become too small to construct meaningful ethno-religious groupings for all ethnic groups.

## Descriptive Results

**Figure 1.**
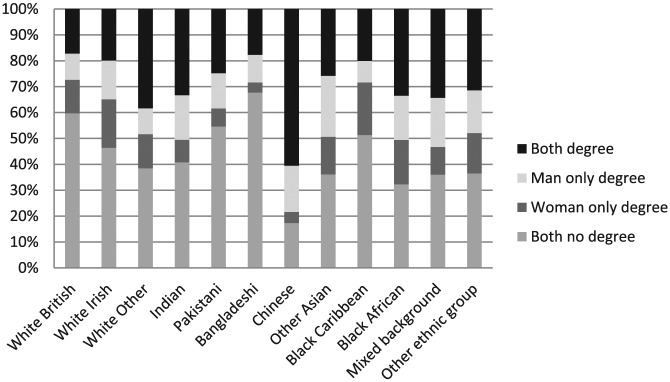
Couples’ joint education status. *Note*: Weighted *N* = 11,866 couples.

**Figure 2.**
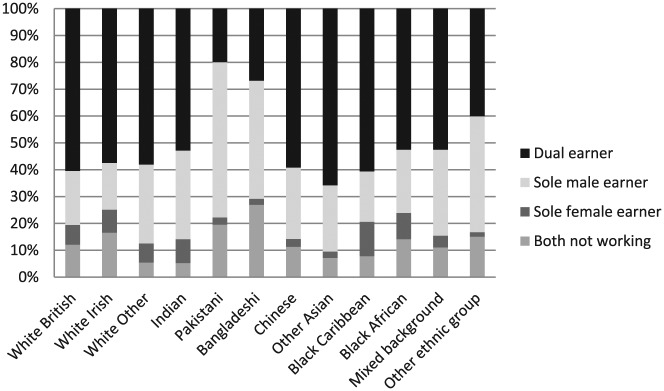
Couples’ joint employment status. *Note*: Weighted *N* = 11,866 couples.

Our hypotheses expect that educational attainment, employment status and gender attitudes differ among ethnic groups and these differences are associated with domestic labour hours and the share of domestic labour within the couple. We expect that the education and employment status not only of each individual but also of their spouse will be significantly associated with housework hours and the share of housework within the couple. Figures 1 and 2 show the joint education status and joint employment status of couples by ethnic group. We see significant variation by ethnic group. Perhaps surprisingly white British couples have one of the highest percentages of neither having a degree (60%), second only to Bangladeshi couples (68%) (Figure 1). Chinese couples are most likely to both have a degree (60%) followed by the white Other group (39%). To some extent these patterns reflect the selection process for immigrants, particularly in recent years where non-EU migration has been controlled through a skilled worker points system.

The majority of couples are dual-earner even though there is considerable variation across ethnic groups (Figure 2). Other Asian couples are most likely to be dual-earner (64%) followed by white British (60%), black Caribbean (60%) and Chinese couples (59%) but with considerable variation in the number of paid work hours within dual-earner couples by ethnic group and gender. Chinese, black African and black Caribbean women in dual-earner couples are most likely to work 30 hours or more at 70 per cent, 68 per cent and 66 per cent respectively. This compares to 65 per cent of white British women, 63 per cent of Indian women, 52 per cent of Pakistani women and 28 per cent of Bangladeshi women.

### Weekly Hours of Housework and Share of Housework

The mean hours spent on housework and the share of housework within couples show that men spend on average fewer than half the hours that women spend on housework, with men having a mean of six hours a week compared to over 14 hours a week for women (Table 2). Men’s share is on average 30 per cent of the total time spent on housework. Across ethnic groups men have fairly similar mean hours spent on housework and the share of housework. Exceptions to this are Other Asian, black Caribbean and black African men who have higher mean housework hours with black Caribbean men having the highest housework share compared to other groups. In contrast Pakistani men report the fewest housework hours and the lowest share of housework of all groups. Women have a greater variation across ethnic groups in the hours spent on housework ranging from a low of 13 hours per week for Chinese and Mixed background women to a high of almost 24 hours per week for Pakistani and Bangladeshi women. This variation is also seen in women’s share of housework ranging from 65 per cent for Mixed background women compared to 83 per cent for Pakistani women.

**Table 2.** Mean hours of housework per week and housework share by gender and ethnicity (married and cohabiting respondents, 16–64 years, waves 2 and 4, *Understanding Society*).

Ethnic group	Men		Women	
	Hours per week Mean (SD)	Housework share Mean (SD)	Hours per week Mean (SD)	Housework share Mean (SD)
White British	6.05 (6.04)	.310 (.224)	14.10 (9.61)	.688 (.227)
White Irish	6.45 (6.31)	.298 (.211)	15.44 (9.58)	.679 (.206)
White Other	5.97 (5.34)	.308 (.207)	14.18 (9.65)	.687 (.224)
Indian	6.67 (6.23)	.252 (.192)	20.22 (10.97)	.756 (.194)
Pakistani	4.85 (6.1)	.176 (.189)	23.80 (14.37)	.834 (.185)
Bangladeshi	6.42 (7.5)	.224 (.214)	23.99 (14.15)	.765 (.210)
Chinese	6.66 (5.13)	.331 (.211)	13.01 (7.52)	.681 (.188)
Other Asian	7.87 (6.17)	.353 (.220)	15.42 (12.1)	.691 (.211)
Black Caribbean	7.12 (6.27)	.379 (.226)	13.43 (10.01)	.671 (.216)
Black African	7.11 (6.62)	.333 (.201)	15.34 (10.24)	.685 (.214)
Mixed background	6.58 (6.39)	.315 (.222)	13.14 (8.64)	.657 (.229)
Other ethnic group	6.35 (5.32)	.295 (.214)	17.41 (10.12)	.736 (.204)
Total	6.10 (6.04)	.308 (.223)	14.44 (9.88)	.691 (.225)

*Note: N* men = 11,866/women = 13,025. Data are weighted.

### Gender-Role Attitudes

**Figure 3.**
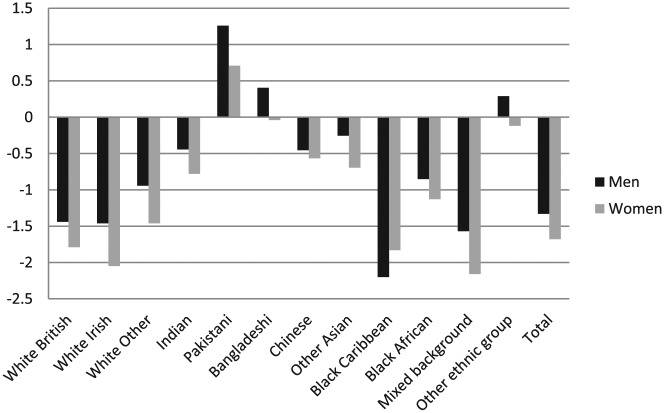
Mean gender-role attitudes score by ethnic group and gender. *Note*: Weighted *N* = 8983 men/10,304 women.

Across all ethnic groups with the exception of black Caribbean, women hold less traditional gender-role attitudes than men in their ethnic group with women having an overall mean score of −1.68 and men a mean score of −1.33 (Figure 3). Interestingly, it is not white British men and women who have the most egalitarian gender attitudes. Women from a Mixed background are less traditional than other women with a mean of −2.16. Only Pakistani women have a positive mean score (.708) indicating more traditional attitudes. Pakistani men have the most traditional attitudes (mean of 1.26). In contrast black Caribbean men hold the least traditional gender-role attitudes of all groups (mean of −2.20). This may reflect a strong history and culture of black Caribbean women being in paid employment making combining family life and paid work the norm for this group.

The descriptive results are consistent with our first hypothesis. There are significant variations in hours of housework, housework share, educational attainment, employment status, work hours and gender-role attitudes by ethnic group and gender. The multivariate analysis in the following section examines the associations among these factors after controlling for individual and household characteristics.

## Multivariate Results

Our second hypothesis is that the gendered domestic division of labour will be associated with educational attainment, employment status and gender attitudes after controlling for individual and household characteristics. Our third hypothesis states that housework hours and housework share are associated significantly with whether individuals are first or second generation immigrants. Table 3, Model 1 predicts the number of housework hours per week by ethnic group where white British is the reference group. Each model is run separately for men and women and Model 2 includes the gender-role attitudes score (included separately because of potential endogeneity). Compared to white British men and controlling for other characteristics, Model 1 shows the only ethnic groups with significantly different housework hours are Indian, Pakistani and Other Asian men. Controlling for all other characteristics stated in the models, Indian and Other Asian men have significantly higher housework hours on average than white British men. These can be interpreted as differences of 48 minutes a week for Indian men and two hours a week for Other Asian men. Holding other factors constant, Pakistani men did 1.3 hours less housework per week than white British men. Taking account of all other characteristics, men whose spouse has a degree-level education increase their housework hours by around 43 minutes a week on average but whether men have a degree is not associated with their housework hours. Having an employed spouse significantly increases men’s housework hours by 1.2 hours a week but set against this, being in paid employment decreases men’s housework hours by over three hours a week. For men, there is no significant association between being born in the UK/arriving before 12 years old and hours of housework.

**Table 3.** Determinants of weekly hours of housework, OLS regression (married and cohabiting respondents, 16–64 years, waves 2 and 4, *Understanding Society*).

	Model 1		Model 2 (with gender attitudes)	
	Men	Women	Men	Women
*White British (ref.)*	–	–	–	–
White Irish	0.160	1.453	−0.114	1.783*
	(0.590)	(0.845)	(0.533)	(0.874)
White Other	0.140	−0.221	−0.139	−0.440
	(0.477)	(0.728)	(0.466)	(0.751)
Indian	0.792*	4.175***	0.942*	3.917***
	(0.395)	(0.632)	(0.422)	(0.668)
Pakistani	−1.258**	4.899***	−0.750	4.731***
	(0.479)	(1.051)	(0.531)	(1.096)
Bangladeshi	0.194	5.805**	−0.242	2.825*
	(0.682)	(1.979)	(0.794)	(1.270)
Chinese	0.940	−0.387	1.225	−1.444
	(0.820)	(1.019)	(0.886)	(1.107)
Other Asian	2.073**	−0.866	1.826*	−0.655
	(0.736)	(1.090)	(0.737)	(1.226)
Black Caribbean	0.735	−0.565	0.491	−0.691
	(0.605)	(0.879)	(0.665)	(0.984)
Black African	0.539	−1.665	0.289	−2.448*
	(0.576)	(0.952)	(0.631)	(0.965)
Mixed background	0.583	0.414	0.763	0.523
	(0.720)	(0.805)	(0.776)	(0.859)
Other ethnic group	0.372	1.173	0.676	0.996
	(0.670)	(1.031)	(0.713)	(1.165)
Has a degree	−0.206	−1.806***	−0.228	−1.619***
	(0.144)	(0.208)	(0.148)	(0.209)
Spouse has a degree	0.713***	−0.883***	0.534***	−0.875***
	(0.150)	(0.213)	(0.154)	(0.216)
In paid employment	−3.383***	−3.865***	−3.319***	−3.626***
	(0.240)	(0.243)	(0.250)	(0.247)
Spouse in paid employment	1.173***	2.149***	0.969***	2.078***
	(0.158)	(0.264)	(0.162)	(0.266)
Born in UK/pre-12 years	0.070	−1.858***	−0.187	−1.893***
	(0.330)	(0.551)	(0.329)	(0.570)
Gender attitudes score	–	–	−0.178***	0.278***
			(0.022)	(0.033)
Controls				
Wave 4 *(ref. wave 2)*	−0.031	−0.752***	−0.040	−0.711***
	(0.093)	(0.139)	(0.097)	(0.144)
Age	0.003	0.165***	0.012	0.160***
	(0.007)	(0.009)	(0.007)	(0.009)
Cohabiting *(ref. married)*	0.539**	−0.060	0.506**	−0.009
	(0.185)	(0.239)	(0.188)	(0.239)
Spouse in same ethnic group	0.082	1.129**	0.127	1.005*
	(0.206)	(0.405)	(0.207)	(0.416)
Log annual household income	−0.157**	−0.457***	−0.154**	−0.432***
	(0.048)	(0.056)	(0.050)	(0.057)
Number of children aged < 16	0.538***	2.703***	0.560***	2.622***
	(0.078)	(0.111)	(0.081)	(0.114)
Constant	8.146***	10.503***	7.800***	11.099***
	(0.645)	(0.895)	(0.666)	(0.932)
Observations	11,866	13,025	10,769	11,923
R-squared	0.061	0.186	0.068	0.194

*Note*: Data are weighted. Robust standard errors in parentheses. ***p < .001; **p < .01; *p < .05.

Differences in sample sizes due to non-response to self-completion questionnaire where the gender attitudes questions were asked. The data are weighted to account for this.

For women there is more variation in housework hours. Indian, Pakistani and Bangladeshi women had significantly higher housework hours than white British women at 4.2, 4.8 and 5.8 hours a week respectively on average (Table 3). This is due in part to the relatively shorter paid working hours of Pakistani and Bangladeshi women where they are in paid employment. There were no significant differences between white British women and women in other ethnic groups. For women, having a degree is associated with reduced housework hours of −1.8 hours a week and having a spouse with a degree also reduces hours of housework by almost one hour (0.9). Being in paid employment has a strong association with reduced hours of housework for women of −3.3 hours a week but having an employed spouse increases housework hours for women (1.1). In contrast to men, women born in the UK or arriving before the age of 12 have significantly lower housework hours (−1.8) than women born outside the UK indicating there are differences between first and second generation women. For women, having a spouse of the same ethnic group increases housework hours and as for men, a higher household income is associated with lower housework hours. Having dependent children is strongly positive with each dependent child increasing women’s housework hours by 2.7 hours a week. Overall our findings show that employment status, education and whether born in the UK or arriving in the UK before 12 are significant determinants of housework hours and housework share. However, ethnic differences in the domestic division of labour remain after controlling for these factors.

In Table 3, Model 2, we test if variations in domestic division of labour can be explained by gender attitudes. Gender-role attitudes are significant in the model for both men and women and in the expected direction. There is a negative relationship for men (i.e. the more traditional men’s gender-role attitudes the lower their housework hours) and a positive relationship for women (i.e. women with more traditional attitudes spend more hours on housework). Pakistani men and Indian, Pakistani and Bangladeshi women continue to have higher housework hours on average compared to white British women even after controlling for gender attitudes, and the magnitude of the ethnicity coefficients does not change much. This suggests that little of the variation in housework hours of women by ethnicity is explained by variations in gender attitudes. For men, the coefficient for Pakistani men becomes insignificant when gender attitudes are included in the model, indicating that the lower participation in housework by Pakistani men compared to white British men is due largely to their more traditional gender attitudes.

When we include interaction effects between ethnic group and education, employment and gender-role attitudes (results not shown but can be made available upon request) we find some differences within groups. Indian men with a degree have significantly higher hours of housework as do Bangladeshi men with a degree, Chinese men whose spouse has a degree, Indian women with a degree and Mixed women whose spouse has a degree. Looking at interactions between ethnic group and employment, white British, other Asian and Other men with an employed spouse have significantly higher housework hours while Pakistani and black Caribbean women with an employed spouse have lower housework hours than those whose spouse is not employed. There are no interaction effects between ethnic group and gender-role attitudes.

### Share of Housework

Table 4 predicts the share of housework for women. Overall a similar picture is found with a positive association for Indian and Pakistani women compared to white British women. The sign for Bangladeshi women is no longer significant. Women who have a degree and those in paid employment have a significantly lower share of housework on average. While the sign is negative for those born in the UK/arriving before 12 years it is not significant in reducing women’s share of housework. Adding gender-role attitudes (Model 2) we see that gender attitudes have a positive association with share of housework; that is, as women’s attitudes become more traditional their share of housework increases even though the size of the effect is small.

**Table 4.** Women’s share of housework, OLS regression (married and cohabiting respondents, 16–64 years, waves 2 and 4, *Understanding Society*).

	Model 1	Model 2 (with gender attitudes)
*White British (ref.)*		
White Irish	0.008	0.017
	(0.020)	(0.020)
White Other	0.011	0.007
	(0.018)	(0.019)
Indian	0.046***	0.047**
	(0.014)	(0.015)
Pakistani	0.093***	0.091***
	(0.014)	(0.016)
Bangladeshi	0.038	0.017
	(0.020)	(0.024)
Chinese	0.015	−0.002
	(0.023)	(0.026)
Other Asian	−0.023	−0.017
	(0.022)	(0.023)
Black Caribbean	−0.005	0.001
	(0.023)	(0.025)
Black African	−0.013	−0.009
	(0.021)	(0.025)
Mixed background	0.003	0.004
	(0.023)	(0.024)
Other ethnic group	0.037	0.038
	(0.024)	(0.026)
Has a degree	−0.058***	−0.052***
	(0.006)	(0.006)
Spouse has a degree	−0.008	−0.008
	(0.006)	(0.006)
In paid employment	−0.075***	−0.071***
	(0.006)	(0.006)
Spouse in paid employment	0.124***	0.123***
	(0.007)	(0.007)
Born in UK/pre-12 years	−0.010	−0.009
	(0.012)	(0.013)
Gender attitudes score	–	0.006***
		(0.001)
*Controls*		
Wave 4 *(ref. wave 2)*	−0.014***	−0.012***
	(0.003)	(0.003)
Age	0.003***	0.003***
	(0.000)	(0.000)
Cohabiting *(ref. married)*	−0.021***	−0.021**
	(0.006)	(0.006)
Spouse in same ethnic group	0.018	0.014
	(0.010)	(0.010)
Log annual household income	−0.011***	−0.011***
	(0.001)	(0.001)
Number of children aged < 16	0.026***	0.024***
	(0.003)	(0.003)
Constant	0.572***	0.583***
	(0.022)	(0.023)
Observations	13,025	11,923
R-squared	0.129	0.138

*Note*: Data are weighted. Robust standard errors in parentheses. ***p < .001; **p < .01; *p < .05.

Differences in sample sizes due to non-response to self-completion questionnaire where the gender attitudes questions were asked. The data are weighted to account for this.

## Discussion and Conclusions

Our first hypothesis expects that the domestic division of labour varies by ethnic group and by gender. We find significant differences between ethnic groups in how couples organise their domestic labour in both the descriptive results and in a multivariate context. In all groups, women spend significantly more hours on housework than men but there is considerable heterogeneity across groups. The share of housework shows less variation with women having an average share of around 70 per cent even though significant differences across ethnic groups remain. Mixed background women have the lowest share of housework (65%) while Pakistani women have the greatest share (83%).

An interesting finding is that it is not necessarily white British couples who are most egalitarian in their division of domestic labour or in their gender-role attitudes. Black Caribbean men have the least traditional gender attitudes of any group, possibly due to matriarchal family structures within Afro-Caribbean cultures. Indian men and Other Asian men spend more hours on housework than their white British counterparts even though Indian, Pakistani and Bangladeshi women spend significantly more time on housework than white British women. It could be as others have suggested (e.g. Sayer and Fine, 2011; Ting et al., 2015), that the definition of what constitutes domestic labour may vary across groups making theoretical approaches used for majority populations and direct comparisons across ethnic groups problematic. Future research could include qualitative approaches exploring whether the definition of housework and the tasks included by individuals when answering these questions are consistent across ethnic groups.

Our second hypothesis expects the relationship between domestic labour and ethnic group would be mediated by educational attainment, employment status and gender attitudes. Our findings support this hypothesis. More egalitarian domestic labour arrangements are significantly associated with having a degree for women as is having a spouse with a degree and more liberal gender attitudes for both men and women. Being in paid employment reduces housework hours on average for men and women but the share of women’s housework is only reduced on average if the woman is employed. Nevertheless ethnic differences in the domestic division of labour cannot be fully explained by these factors. Indian, Pakistani and Bangladeshi women still have significantly higher housework hours after taking these factors into account suggesting there may be cultural or other unobserved differences not available in the data.

Our third hypothesis expects domestic division of labour arrangement differs between those who were born in the UK or arrived in the UK before age 12 and first generation immigrants. We find housework hours reduce for second and one-and-a-half generation women but there are no significant differences for men. While there are some indications of differences between first and second generation women it may take considerable time for norms of behaviour to change.

In sum, concurring with resource bargaining theory and time availability perspectives, our study shows that gender inequality in the domestic division of labour can be lessened by higher educational attainment and women being in employment even though the intersection between gender and ethnicity and domestic labour varies across ethnic groups. The intersectionality between gender and ethnicity in terms of housework arrangement is also mediated to some degree by gender attitudes.

The analysis provides the first quantitative evidence on the intersections between gender, ethnicity and the domestic division of labour for the UK. It could be argued that survey data are a rather blunt instrument for fully understanding the complexities of the domestic division of labour and the nuances of negotiations between couple members. While we cannot provide any causal explanation for why we find differences in patterns of domestic labour across ethnic groups, the analysis shows clear associations between gender and ethnicity, education levels, employment status and gender attitudes. The multivariate analysis identifies both differences and similarities between ethnic groups and suggests theoretical perspectives focusing on gender alone may be inadequate when examining the intersections between gender, ethnicity and domestic labour. Despite the large sample size for the survey, our analysis is limited by small numbers of cases for some ethnic groups. This limitation can be overcome when more waves of the *Understanding Society* data become available. Future studies should endeavour to investigate the mechanisms that explain the variations in the gender division of labour among different ethnic groups and the complex intersections between gender, ethnicity, socio-economic position and domestic labour that determine gender inequalities.
